# Cerebral Air Embolism: A Case of a Rare Transthoracic Needle Biopsy Complication

**DOI:** 10.7759/cureus.35203

**Published:** 2023-02-20

**Authors:** André Santos, Catarina Almeida, Lenea M Porto, Pedro D Fernandes, João P Silva

**Affiliations:** 1 Department of Internal Medicine, Centro Hospitalar Tondela Viseu, Viseu, PRT; 2 Department of Pulmonology, Centro Hospitalar Tondela Viseu, Viseu, PRT

**Keywords:** lung biopsy, cerebral air emboli, cerebrovascular accident (stroke), vascular air embolism, transthoracic needle biopsy

## Abstract

Transthoracic needle biopsy (TNB) is a fundamental procedure in the diagnosis of a wide spectrum of thoracic diseases replacing more invasive surgical procedures. The procedure may be performed with computed tomography (CT) or ultrasound imaging guidance, with CT being the more commonly utilized. Although less invasive than surgery, there is still a complication risk associated with this procedure. These can be local such as pneumothorax, parenchymal hemorrhage, tumor seeding, and hemoptysis, or systemic such as air embolism. The authors report a case of cerebral circulation air embolism as a complication of TNB in a 54-year-old male with suspected lung tumor followed by a brief review of the current literature.

## Introduction

Percutaneous transthoracic needle biopsy (TNB) is a safe, minimally invasive, indispensable tool in the diagnosis of thoracic lesions [1]. Typically used to evaluate indeterminate pulmonary nodes or masses, especially solitary pulmonary lesions, TNB can efficiently provide successful histologic or cytologic diagnoses while reducing the need for more invasive procedures, which in the end reduces healthcare costs and benefits the patient due to shorter hospitalization times. A meta-analysis conducted by Schreiber and McCrory revealed that the overall sensitivity for biopsy of pulmonary lesions was 90% (95%CI 0.88-0.92) [2]. Usually, TNB is assisted by ultrasound or CT scan guidance with low complication rates reported when performed by appropriately trained and experienced physicians. Regardless of its safety, there are some complications directly related to this procedure described in the literature.

The most reported complication is certainly pneumothorax (23.3% of patients) [2]. It is well known and described in the literature that chronic obstructive pulmonary disease (COPD) is one of the most significant risk factors for pneumothorax, likely related to the presence of emphysema in these patients. A retrospective study conducted by Vachani et al. in 2022 reported that inpatient history of COPD, prior bronchoscopy, and prior lung cancer screening were associated with increased risk of pneumothorax while younger age and pleural effusion were associated with a decreased likelihood of the event [3,4]. Another study conducted by Khan et al. reported that small lesion size and increased depth of the lesion from the skin (> 4 cm) were also associated with an increased risk of pneumothorax [5]. Parenchymal hemorrhage is the second most reported complication found in the literature (around 4% of patients) [6]. The occurrence of significant hemothorax is extremely rare. A study conducted by Tomiyama et al. with data from 9783 biopsies revealed an incidence of 0.092% of hemothorax events [6]. Like what was described with pneumothorax, small lesion size and greater lesion depth were associated with a higher risk of bleeding. According to Heyer et al., hemorrhage may also be related to the presence of emphysema reported in CT scans, probably due to the lack of effective tamponade by adjacent tissue [7]. Patients with pulmonary arterial hypertension (PAH) have also a higher risk for hemorrhage due to the increased vascular pressures [1]. It should be held to account that TNB is contraindicated in patients with known bleeding diathesis or under anticoagulant or antiplatelet medication. These medications should be appropriately withheld depending on their half-life. Major central vessels should be avoided by careful procedure planning, especially systemic arteries like internal mammary, subclavian, and axillary arteries.

Systemic air embolism and tumor seeding are rarely reported [6]. Although potentially fatal, air embolism is extremely rare. A study conducted by Hiraki et al. reported only four cases of accidental air embolism in 1010 procedures performed between April 1999 to December 2006 [8]. A more recent study published in the European Radiology Journal in 2022 revealed an overall incidence of around 0.23% [9]. Despite its low incidence, the risk of air embolism is thought to be related to vasculitis, cavitary or cystic lesions, and positive pressure mechanical ventilation (PPMV) [9]. Tumor seeding along the needle pathway is even less frequently reported. Some studies report incidences between 0.012% and 0.061% [10]. Commonly affected sites are the pleura or chest wall but systemic metastization may occur if a vessel is punctured along the pathway. No specific risk factors have been identified for this complication [11]. Careful pre-biopsy planning and appropriate postprocedural care are fundamental to preventing or minimizing most of the potential complications.

## Case presentation

**Figure 1:**
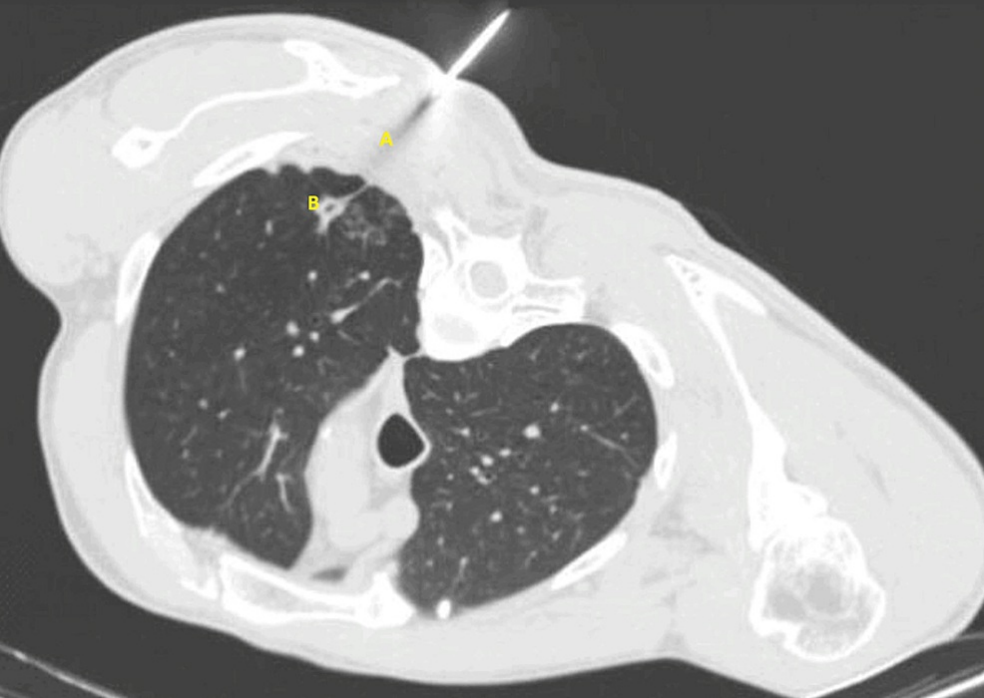
Transthoracic needle biopsy axial CT scan image showing posterior transthoracic needle biopsy with proper placement of introducer needle at edge of left upper lobe nodule (B) and the resulting pathway (A)

Here, we describe a case of a 54-year-old heavy smoker man (> 60 pack-years) with known emphysema and two suspicious left superior pulmonary lobe nodes. The patient was admitted to the pulmonology ward to perform a diagnostic TNB of a cavitated spiculated nodule located at the left superior lobe (Figure 1). The procedure was conducted by our intervention radiologist and expert pulmonologist in the procedures operating room from our department of pulmonology. To perform the biopsy an automatic Tru-Cut® biopsy device (Merit Medical Systems, Inc., South Jordan, Utah, United States) with a 14G needle was used.

The patient was placed in a supine position and was instructed to avoid deep breaths or talking during the procedure. The patient was able to hold his breath every time he was asked to (during the needle insertion, tissue collection, and needle removal). No unexpected events were reported during the procedure.

A few minutes after the procedure the patient presented a transient loss of consciousness followed by speech difficulty and apparent loss of strength in the right arm. The intrahospital stroke emergency team was promptly activated. The first observation of the patient revealed global aphasia (National Institutes of Health Stroke Scale (NIHSS) = 3), right arm paresis grade 2 (NIHSS = 3), and facial palsy with right upper motor neuron (UMN) pattern (NIHSS=2), totaling 8 points at the NIHSS. The patient’s blood glucose level was 99mg/dL and blood pressure was 130/90 mmHg with a heart rate of 97 bpm. The peripheral O2 saturation was 98% (FiO2=21%). The diagnostic of acute stroke was assumed.

**Figure 2:**
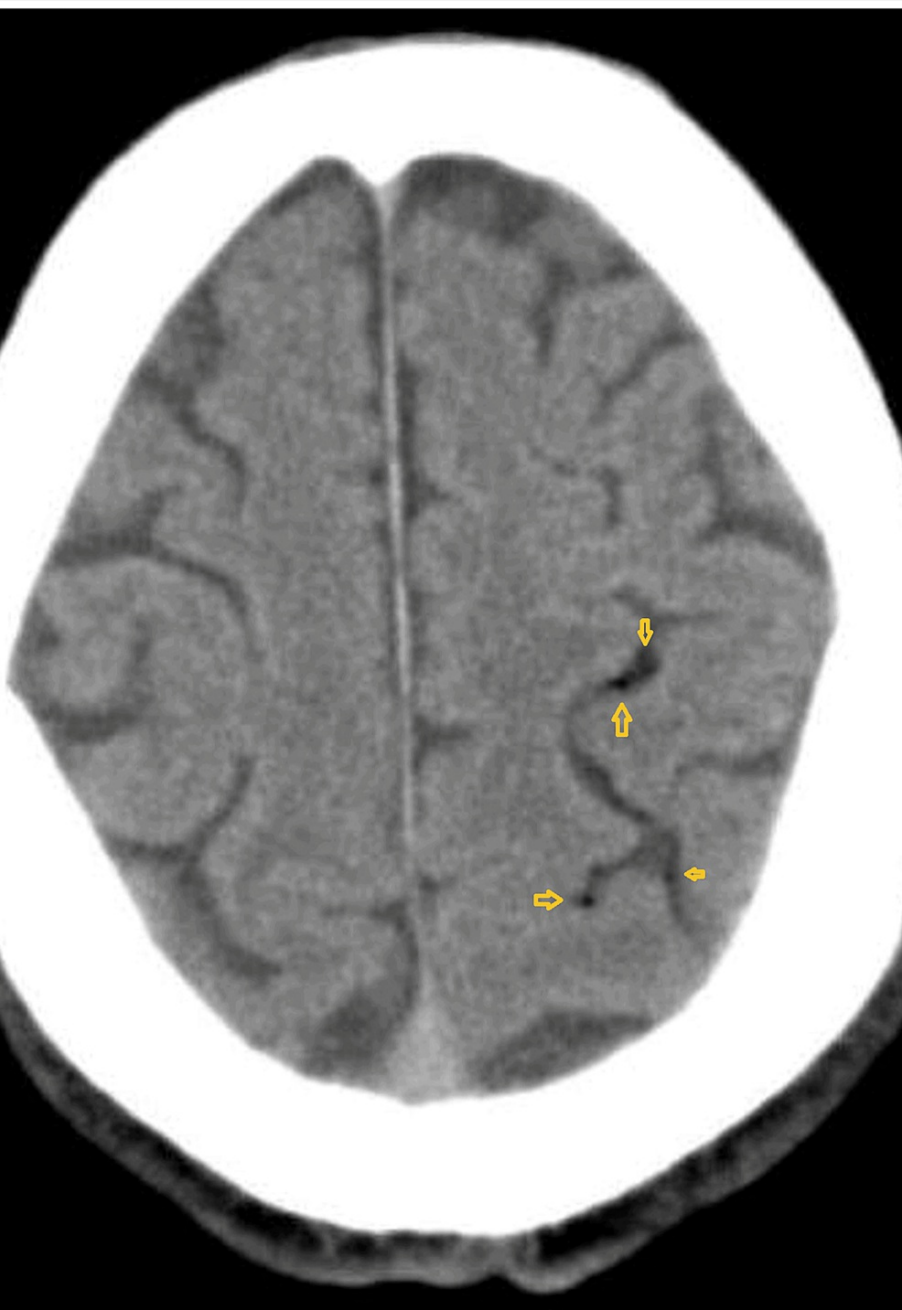
Axial Brain CT scan which revealed small low-density spots at the left parieto-occipital region suggestive of subarachnoid air bubbles (arrows) due to air embolism

Around 20 minutes after the onset of symptoms, the patient underwent a non-contrast brain CT scan, which revealed small amounts of subarachnoid air bubbles at the left parieto-occipital region suggestive of air embolism (Figure 2). After the initial CT scan, a brain CT angiography was also performed, with the latter showing no signs of significant thrombi or strictures on the main neck and intracerebral arterial vessels. The patient was immediately taken to the acute stroke unit where he was started on oxygen therapy administered by a high flux high concentration mask. A hyperbaric chamber center was contacted in order to transfer the patient for further treatment but, before proceeding to the transport, the patient achieved a complete resolution of the initial neurological symptoms. Another brain CT scan was done and revealed no signs of air at the previous location as no other lesions. The biopsy histologic study revealed no malignancy and the patient proceeded to surgical nodule resection after four months, which also ruled out malignancy. Despite the described complication, the patient had a complete resolution of the neurological symptoms, and no further complications were reported.

## Discussion

Percutaneous TNB is a frequent procedure that is considered a standard first method for the diagnosis of pulmonary lesions. With an estimated occurrence < 0.3%, air embolism is an extremely rare but potentially fatal complication of TNB [2,6,11]. Two mechanisms may be involved in the creation of an air embolus capable of traveling via the left heart into the systemic arterial circulation. Air bubbles may enter the pulmonary veins by the accidental placement of the needle tip within a pulmonary vein, followed by air injection into the needle; another mechanism consists in the creation of a fistula between a bronchus and an adjacent pulmonary vein by the inadvertent placement of the needle through both structures, allowing passage of air into the pulmonary vein. It’s not difficult to understand these mechanisms considering that pulmonary vein pressure is normally around 10 cmH2O [12].

Severe conditions may develop once air bubbles enter the arterial circulation. Even when only small amounts of gas enter the arterial system, the transport of gas bubbles into end arteries will cause the occlusion of these vessels. Although any artery may be occluded by gas bubbles, when it involves either the coronary arteries (coronary gas embolism) or the brain nutritive arteries (cerebral arterial gas embolism), the outcome may be especially serious, possibly with a fatal course of events due to the high vulnerability of these organs to short periods of hypoxia [13]. When air emboli enter the cerebral circulation, they cause pathologic changes by reduced perfusion distal to the obstruction and by the consequent inflammatory mechanisms that develop in response to the air bubble. Therefore, arterial air emboli may cause myocardial infarction, cerebral infarction, and even death.

The current literature seems to support the idea that the placement of patients in the Trendelenburg position is recommended as soon as possible when air embolus is suspected [14]. Contrary to the previous statement, a review by Muth et al. reveals that a supine horizontal position may be as effective as Trendelenburg to minimize the dispersion of air emboli into the brain [15]. Once air embolism is suspected, immediate additional measures should be taken. Rapid volume expansion with IV fluids is indicated to elevate venous pressure in order to reduce the continued entry of gas into the venous circulation. Usually, oxygenation is only possible with low-pressure high oxygen concentration masks delivering up to a fraction of inspired oxygen (FiO2) of 100%. If a hyperbaric oxygen chamber is available, it could also help by increasing the gradient for of nitrogen removal from the air bubble. It is stated by Boyle's law that the size of gas bubbles in a liquid tends to decrease with increased pressure, which is the basis for using hyperbaric medicine in the treatment of this complication [16].

Regarding the current case, it is important to mention that our hospital center has a lot of experience performing TNB and this was the first reported case of air embolism. We believe that the existence of a dedicated stroke team and a good intrahospital stroke fast track was preponderant for the resolution of the event without permanent sequelae even if only atmospheric pressure oxygen sources were available. Regarding the procedure itself, in our center, some preventive measures are usually taken to prevent these events. Patients never undergo lung biopsy while in an upright position. The needle introducer should always be occluded (by the inner stylet or the operator's finger). The patient should be instructed to avoid moving, taking deep breaths, or coughing during the biopsy. It is vital that the patients understand that they need to comply whenever asked to hold their breath to ensure that the needle is getting tissue from the right area. Patients on mechanical ventilation should be put under expiratory pause during needle manipulation. Regarding cerebral air embolism, we believe that the supine position is preferable to the Trendelenburg position, as the latter may aggravate cerebral edema and lead to generalized seizures.

## Conclusions

The presented patient was a heavy smoker, which is a common risk factor for lung cancer and emphysema. Emphysema is one of the major risk factors for TNB complications, especially pneumothorax, which was the most expected complication. Despite the fact that the procedure protocol was correctly followed, the patient presented one of the most severe yet extremely rare complications described for this procedure. Fortunately, the identification and treatment of this severe condition were achieved in time and the patient had a full recovery with an excellent outcome when we consider that malignancy was ruled out. Even with a highly trained team and careful procedure preparation, it’s virtually impossible to predict and avoid complications in this kind of procedure considering its invasiveness and sensitivity of chest structures and keeping in mind that usually the patients who undergo these technics present specific characteristics that make them more vulnerable for complications.

When we consider specifically arterial air embolisms, the available information in the literature is poor due to the rareness of these events. Nevertheless, it should be highlighted that following the available recommendations is vital to minimize these complications and, in case of an event, fast diagnosis and treatment are fundamental to achieve a good outcome.
